# Fragmented care in lupus: Patient experiences and insights

**DOI:** 10.4102/ajod.v14i0.1562

**Published:** 2025-01-28

**Authors:** Armand Bam

**Affiliations:** 1 Stellenbosch Business School, Faculty of Economics and Management Science, Stellenbosch University, Cape Town, South Africa

**Keywords:** systemic lupus erythematosus, chronic illness management, multidisciplinary care, care fragmentation, patient-centred, interprofessional collaboration, team dynamics, integrated care

## Abstract

**Background:**

Systemic lupus erythematosus (SLE) is a complex autoimmune disease requiring interprofessional collaborative care because of its varied manifestations. This study explores the experiences of individuals living with SLE regarding the communication and collaboration among their healthcare providers.

**Objectives:**

This study aimed to explore the communication dynamics that shape treatment experiences and well-being in SLE care.

**Method:**

A qualitative multiple case study design was used, with thematic analysis of semi-structured interviews from six people living with SLE.

**Results:**

Three primary themes emerged: the necessity of cohesive healthcare teams, the centrality of client-centred care and the significant challenges posed by fragmented healthcare systems.

**Conclusion:**

The study highlights the need for integrated care models to enhance communication and coordination among healthcare providers.

**Contribution:**

This research adds empirical insights into the communication dynamics within medical teams managing SLE, advocating for client-centred and systemic improvements in care coordination.

## Introduction

Systemic lupus erythematosus (SLE) is a complex autoimmune disease that can present with a wide range of clinical manifestations, making its diagnosis and management challenging. This complexity requires not only precise diagnosis but also comprehensive multidisciplinary care to manage the disease effectively. Despite advances in treatment and a deeper understanding of SLE’s underlying mechanisms, people living with SLE continue to experience high morbidity and mortality rates because of complications such as major organ failure, infections and cardiovascular disease. Effective communication and coordination among healthcare providers are therefore critical for improved patient outcomes.

However, while patient or client-centred care and shared decision-making (SDM) are increasingly recognised as essential in chronic disease management, there remains a significant gap in understanding the specific dynamics of communication within medical teams managing SLE. This study seeks to address this gap by exploring the experiences of people with SLE regarding the communication dynamics among the professionals who provide their care. By investigating the experiences of individuals living with SLE regarding the communication and collaboration among their healthcare providers, the research aims to provide new insights into improving chronic disease management through enhanced interprofessional collaboration.

## Literature review

The management of SLE has evolved significantly in recent years ushering in new treatments and an improved understanding of the disease’s underlying mechanisms (Morand et al. 2023). Globally, recommendations for SLE management exist, emphasising an integrated approach to care to avoid fragmentation (Bertsias et al. 2008). Despite these advancements, patients with SLE continue to face high morbidity and mortality rates, with major organ failure, infection and cardiovascular disease being significant challenges (Katarzyna et al. 2023). The clinical heterogeneity of SLE, with diverse phenotypes stemming from different molecular pathologies, contributes to the difficulties in standardising treatment approaches (Morand et al. 2023). Therefore, the effective communication and collaboration among healthcare providers from various specialities are essential to address the multifaceted needs of SLE patients.

Patient-centred care and client-centred care share essential principles. Both models prioritise the individual’s needs, autonomy and involvement in decision-making, focusing on responsive care that aligns with personal values (Epstein & Street 2011; Mead & Bower 2000). While ‘patient-centred care’ is more common in clinical contexts, emphasising medical outcomes, ‘client-centred care’ is often used in social work and mental health, reflecting the active role of individuals in shaping their care plans (Kitson et al. 2013; Morgan & Yoder 2012). These models reflect a convergence in healthcare goals, with both frameworks promoting empowerment, autonomy and collaborative care, which is well suited to managing complex chronic conditions like lupus (Fix et al. 2018; McCormack & McCance 2006).

Studies have highlighted the importance of patient and client-centred care and SDM in the management of chronic conditions like SLE (Politi et al. 2013; Stiggelbout, Pieterse & De Haes 2015). However, there remains a paucity of research on the specific communication dynamics within medical teams caring for individuals living with SLE. Recognising the need to better understand this critical aspect of SLE management, the present study aimed to explore the experiences and perspectives of people living with SLE regarding the communication and collaboration among the medical professionals involved in their care (Spath et al. 2011; Thorne et al. 2004; Villalobos et al. 2017).

### Client-centred care and chronic illness management

Client-centred care is a fundamental principle in managing chronic illnesses (Bauman, Fardy & Harris 2003; Mirzaei et al. 2013). By focusing on the patient’s experience and involving them in decision-making processes, healthcare providers can ensure that care plans are tailored to the individual’s needs and preferences for better health outcomes (Cornet et al. 2021; Williams, Ortiz & Browne 2014). Joughin et al. (2021) highlight the importance of feedback-seeking behaviour in enhancing client-centredness applicable to healthcare where feedback literacy can improve patient outcomes. They suggest that continuous feedback can help service providers adjust their strategies to better meet their client’s needs. This is supported by others who emphasise the role of feedback in the academic socialisation of students within healthcare, where continuous learning and adaptation are essential for delivering client-centred care (Kim 2018; Mohd Noor et al. 2024; Tripodi et al. 2021).

Shared decision-making is central to client-centred care. It involves patients and healthcare providers collaborating to make healthcare decisions that align with the patients’ values, preferences and lifestyle (Légaré & Witteman 2013; Politi et al. 2013; Stiggelbout et al. 2015). Shared decision-making improves patient satisfaction, adherence to treatment and health outcomes (Rosa et al. 2023; Van der Horst et al. 2023). It is particularly important for people living with SLE because of the complexity and variability of the disease, which requires personalised treatment plans that consider their unique circumstances (Duda-Sikuła & Kurpas 2023; Montori et al. 2023).

The use of patient-reported outcome measures (PROMs) is another important aspect of client-centred care. Patient-reported outcome measures allow patients to provide direct feedback on their health status, treatment effectiveness and quality of life (Adeghe, Okolo & Ojeyinka 2024). Integrating PROMs into clinical practice improves patient-provider communication and enhances the overall quality of care where this information can be used to tailor care plans and ensure that they address the specific needs and concerns of the patient (Makhni & Hennekes 2023).

Cultural competence also plays a significant role in delivering client-centred care. Healthcare providers must be aware of and sensitive to the cultural, social and economic factors that influence patients’ health beliefs and behaviours (Taylan & Weber 2023). Cultural competence training can help healthcare providers understand and respect the diverse backgrounds of their patients, leading to more effective and personalised care. Cultural competence is therefore essential for reducing health disparities and improving health outcomes for minority populations (Reid et al. 2023; Yalavarthi et al. 2023).

In addition, the role of technology in enhancing client-centred care is increasingly being recognised (Bertolazzi, Quaglia & Bongelli 2024). Tools such as patient portals, mobile health apps and telehealth services can facilitate better communication and engagement between patients and healthcare providers. The use of telehealth services has been shown to improve patient satisfaction and access to care, particularly for those with chronic conditions like SLE. These technologies enable patients to actively participate in their care, access their health information and communicate with their healthcare team from the convenience of their homes (Bhatt & Chakraborty 2023). The technologies place the patient at the centre enabling them to monitor their health, access resources and communicate with their healthcare providers, contributing to better management of their condition and improved health outcomes (Howarth et al. 2018).

Another important aspect of client-centred care is the emphasis on holistic approaches that consider the physical, emotional and social dimensions of health. Holistic care approaches, which integrate mental health support, social services and community resources, can significantly enhance the well-being of patients with chronic illnesses (Cano et al. 2023). This approach aligns with the biopsychosocial model of health, which recognises that health outcomes are influenced by a combination of biological, psychological and social factors (Hunt 2024).

### Collaborative teams in healthcare

The importance of collaboration to ensure that patients receive comprehensive and coordinated care is well documented (Bauman et al. 2003; Soukup et al. 2018). Effective teamwork is therefore essential in managing chronic illnesses like SLE. The role of multidisciplinary teams (MDTs) (Wagner 2000), interdisciplinary teams (IDTs) (Boult & Karm 2008) and interprofessional collaborative practice (IPCP) (Hall 2005; Keshmiri & Barghi 2021; WHO 2010) in healthcare settings has been extensively studied. Multidisciplinary teams enable the sharing of diverse perspectives when multiple disciplines come together contributing independently to a common problem, maintaining its methods and perspectives and addressing aspects of the problem within its boundaries (Farina et al. 2022). Interdisciplinary teams, on the other hand, seek to collaborate integrating knowledge and methods from their respective disciplines. This integration should result in a cohesive understanding or solution. The focus here is the synthesis and interaction across disciplinary boundaries, creating a unified approach to problem-solving (Boult & Karm 2008). Moreover, IPCP involves professionals from varying fields using their distinct expertise to improve outcomes. This approach emphasises cooperation and teamwork with clear professional roles and boundaries where there is a collective contribution to a shared goal without the blending of professional identities (D’Amour & Oandasan 2005).

While each approach is crucial for providing comprehensive care, the effectiveness of these teams often hinges on strong leadership to ensure alignment towards common goals. Significantly, leadership styles that promote open communication, mutual respect and SDM have been shown to enhance team performance and patient care (Salas, Zajac & Marlow 2018). The importance of leadership in fostering effective teamwork is essential for improved patient outcomes. The presence of strong leadership can help navigate the challenges of managing chronic illnesses, ensuring team members are aligned towards common goals (Al-Sawai 2013). This is particularly relevant in healthcare settings where the complexity of managing chronic illnesses requires coordinated efforts from various healthcare professionals.

The role of training and development in enhancing teamwork cannot be overlooked. Training programmes that focus on team-building skills, communication and conflict resolution can significantly improve team performance. According to Gittell, Seidner and Wimbush (2010), relational coordination training, which emphasises the importance of communication and relationships among team members, can lead to improved patient outcomes and higher job satisfaction among healthcare professionals. This highlights the need for ongoing training and professional development to maintain high standards of teamwork in healthcare settings (Hossny, Gabra & Hussien 2022). The impact of organisational culture on training and teamwork is another critical area of exploration where it has been shown that organisations that value collaboration and continuous learning can foster effective teamwork and improve patient outcomes (Rosen et al. 2018).

The integration of technology in teamwork is also a significant trend. Tools like electronic health records (EHRs), telemedicine and collaborative platforms can enhance communication and coordination among team members, while the use of EHRs improved the efficiency of information sharing and decision-making in healthcare teams, leading to better patient outcomes (Konnoth & Scheffler 2020). Similarly, telemedicine has been shown to facilitate remote collaboration and consultation, which is particularly beneficial in managing chronic conditions that require ongoing monitoring and intervention (Bashshur et al. 2014; Petruzzi & De Benedittis 2016).

### Healthcare complexity and managing systemic lupus erythematosus

Effective management of SLE frequently necessitates involving professionals from various fields (Abu Bakar et al. 2020). Coordinating care among multiple professionals, each with their own expertise and communication styles, can be challenging, especially within healthcare systems facing resource constraints and siloed departments. For example, a patient with SLE may require input from a rheumatologist, nephrologist, dermatologist and psychiatrist, depending on their individual disease manifestations (Bertsias et al. 2008). Breakdowns in communication or coordination among these professionals can lead to fragmented care, delays in treatment and poorer patient outcomes (Hossny et al. 2022). Research suggests that integrated care models, characterised by effective communication and collaboration among healthcare providers, are associated with improved outcomes for patients with chronic illnesses, including SLE (Makhni & Hennekes 2023).

The multifaceted nature of SLE presents considerable challenges for healthcare professionals, particularly within the context of complex and demanding work environments. The highly variable disease course of SLE, coupled with the potential for multiple organ involvement, places a significant cognitive load on professionals (Patel et al. 2018). They must process large amounts of information, often ambiguous and rapidly changing, to make timely and accurate diagnoses and treatment decisions. Here, differentiating between a lupus flare and an infection, both of which can present with similar symptoms, requires careful clinical judgement and often necessitates further investigations (Morand et al. 2023). This cognitive burden can be exacerbated by workplace stressors such as time pressures, high patient volumes and administrative burdens, potentially increasing the risk of burnout and medical errors (Gandhi et al. 2023). Moreover, studies have shown a correlation between physician burnout and decreased quality of care, including increased medical errors and reduced patient satisfaction (Gandhi et al. 2023).

South Africa’s healthcare system has been shaped by its apartheid past and continues to be plagued by resource constraints (Maphumulo & Bhengu 2019). Twenty years post-democracy, the annual per capita expenditure on health was estimated at $140 in the public sector staffed by 30% of the doctors and serving 84% of the national population, while the per capita spend in the private sector was estimated at $1400 where 70% of the doctors were serving 16% (8 million) of the population (Mayosi & Benatar 2014). The latest data shows that little has changed as 84% of the population remains uninsured and reliant on the public healthcare system (Statistics South Africa 2022). This poses significant challenges to the delivery and access to optimal client care, potentially exacerbating existing disparities in health outcomes for people with SLE (Harris et al. 2011). Acknowledging scarcity in public healthcare systems compared to private is crucial for cost containment and fair allocation of resources.

Limited access to professionals, diagnostic testing and novel therapies, often compounded by long wait times for appointments and procedures, can delay diagnosis, hinder timely treatment initiation and negatively impact disease management for patients with SLE (Fradgley, Paul & Bryant 2015; Johnston, Wen & Joynt Maddox 2019). Moreover, the trade-off between hospital efficiency and mortality rates, suggesting that pressure to reduce costs can sometimes compromise patient outcomes (Ezeonwu 2018). Insufficient staffing levels and high patient-to-provider ratios can also lead to rushed consultations, inadequate patient education and diminished continuity of care, ultimately affecting patient satisfaction and adherence to treatment plans. These capacity constraints influence patient allocation and impact the intensity of treatment received. Addressing resource allocation disparities and ensuring adequate funding for SLE care are therefore crucial to improving patient outcomes and reducing health inequities (Cope et al. 2019).

While each professional contributes unique expertise, a lack of coordinated communication can lead to conflicting treatment plans, medication errors and a disjointed patient experience (Van der Horst et al. 2023). To address this challenge, the integration of EHRs and other health information technology tools can play a pivotal role in facilitating data sharing, care coordination and decision making (Bhatt & Chakraborty 2023). Furthermore, professionals may face challenges related to workplace culture and support systems. A lack of understanding or awareness of SLE among colleagues can lead to inadequate support for professionals managing complex cases (Morand et al. 2023). Additionally, organisational factors such as limited access to continuing education opportunities or inflexible work schedules can hinder professionals’ ability to stay updated on the latest SLE research and best practices (Reid et al. 2023). Addressing these workplace-related challenges is crucial to enhancing the capacity of healthcare professionals to provide high-quality care for individuals living with SLE.

## Research methods and design

This overarching study employed a multiple case study design to explore the experiences of individuals living with SLE in South Africa. A multiple case study design allowed for in-depth exploration of a phenomenon across different contexts, providing robust and reliable evidence through the analysis of similarities and differences between cases. This approach was chosen to capture the complexities and nuances of the participants’ experiences in various employment settings (Baxter & Jack 2015). The unit of analysis was the individual living with SLE, focusing on how they navigated treatment, faced challenges and coped with their condition (Eisenhardt 1989). Each case considered the nature of the participants’ treatment, disability and the social dynamics within their described medical settings. The study examined participants’ experiences of receiving medical treatment from the time of their SLE diagnosis to the present, within the geographical context of South Africa, which influences local healthcare systems, treatment access and societal attitudes towards chronic illness and disability.

### Participants

The study included six participants diagnosed with SLE. Participants were selected using purposive sampling to ensure they could provide detailed and relevant insights into the research question. Each participant underwent two in-depth interviews, each lasting between 1 and 2 h, allowing for comprehensive data collection. All participants’ identities were protected with pseudonyms.

#### Case profiles

Ross was 41 years and was diagnosed at 28 years old. His diagnosis occurred during a period of temporary unemployment affecting his health insurance. He had sought care at a public hospital initially; but because of the extended waiting period and witnessing the death of a fellow patient in the corridors, he left without consulting a professional. Subsequently, his diagnosis occurred in a private healthcare facility, and he has continued to make use of private healthcare services. Carmen was a 57-year-old woman and was diagnosed at 37 years. Her initial diagnosis was incidental because of an unrelated surgery. She subsequently enrolled in a lupus clinic in a public healthcare facility because of a lack of medical insurance and spent 14 years in the programme until she could afford private healthcare. Julie was 53 years old and was diagnosed at the age of 28 years. She has always had access to healthcare insurance, which has allowed her to be consistently under the care of private healthcare professionals from a young age. Esther was 35 years old and was diagnosed at 21 years of age. She considered herself privileged always having had access to private healthcare. Esther acknowledged that this access to medical insurance was essential to afford the best possible treatment. Erica was 43 years old and received a diagnosis at the age of 36 years. She has always made use of private healthcare. Keisha was 38 years old and was diagnosed at the age of 23 years and has only been within the public healthcare system. Because of extensive periods of unemployment and low paying jobs, she has not been able to afford healthcare insurance.

### Data collection

Semi-structured interviews, guided by an interview schedule, were used to collect data. This approach allowed for flexibility in probing for deeper understanding while maintaining consistency across interviews (Rabionet 2011). Interviews were audio recorded and then transcribed verbatim for accuracy in data analysis. Field notes were also captured of the non-verbal cues for additional contextual information that would not be captured through the transcription process (Jack 2008).

### Data analysis

Data were analysed using a thematic analysis approach following Braun and Clarke’s (2006) six-step method. Additionally, cross-case synthesis was employed to analyse data across multiple cases, identifying patterns and themes that emerged within and between cases (Jack 2008; Yin 2013). The steps included familiarisation with the data, where transcripts were read multiple times to become deeply familiar with the content. Interesting features of the data were coded systematically across the entire data set. Codes were collated into potential sub-themes, gathering all data relevant to each sub-theme. Sub-themes were then grouped into the overarching themes that were then checked to ensure they worked in relation to the coded extracts and the entire data set (Hsieh & Shannon 2005). Each theme was refined, and clear definitions and names were developed and illustrated in Table 1: Themes, Sub-themes and Codes. Finally, a report was produced, providing vivid examples and relating back to the research question and literature. Data were managed using ATLAS.ti software, which facilitated the coding and organisation of data, ensuring a systematic approach to theme development and cross-case analysis (Yin 2013).

**TABLE 1:** Themes, sub-themes and codes.

Theme	Sub-theme	Code	Example
Collaborative care	Patient-constructed support networks	Team Building	‘I’ve known I need people around me. I don’t have all the answers.’ (Ross, Participant 1, Male, 41 years)
	Importance of interprofessional networks	Practitioner Compatibility	‘You don’t need that. There are other doctors that are great …’ (Esther, Participant 4, Female, 35 years)
	Value of practitioner’s SLE expertise	Specialist Knowledge	‘It’s very hard to find the right doctors…A lot of people think they know something …’ (Esther, Participant 4, Female, 35 years)
	Impact of proximity for better collaboration and service integration	Geographic Proximity	‘It was good when all of them were in one hospital … But now I have to go to different hospitals.’ (Erica, Participant 5, Female, 43 years)
	Patient role in educating practitioners and advocating for better understanding	Patient Advocacy	‘I put up fliers and asked for 10 minutes … explained Lupus is an autoimmune disease.’ (Erica, Participant 5, Female, 43 years)
Patient-centredness	Use of technology for remote access to healthcare	Accessibility and Convenience	‘I would have WhatsApp calls, video calls … it’s easy for me not to leave home.’ (Julie, Participant 3, Female, 53 years)
	Importance of transparency and empathy in communication	Empathetic Communication	‘Don’t get your hopes up … This is going to be a long journey.’ (Julie, Participant 3, Female, 53 years)
	Self-awareness in recognising symptom patterns and managing proactive healthcare	Symptom Monitoring	‘I say, “Something’s brewing” … by week 3 I say, “Now I’m coming, I need to see you.’’’ (Julie, Participant 3, Female, 53 years)
	Empowering patients through knowledge for self-advocacy	Patient Autonomy	‘I will question and you need to give me answers that I need.’ (Julie, Participant 3, Female, 53 years)
	Navigating healthcare system challenges, especially during critical events like COVID-19	Resilience	‘Covid hit … Luckily I found a rheumatologist who figured out after three months …’ (Esther, Participant 4, Female, 35 years)
Fragmentation challenges	Need for better communication across healthcare professionals	Interdisciplinary Communication	‘I struggle most with [*getting*] all of my medical professionals to communicate with one another.’ (Keisha, Participant 6, Female, 38 years)
	Conflicts because of uncoordinated care among different specialists	Contradictory Treatments	‘Dr A says to remove the stents; Dr B says they must stay in …’ (Erica, Participant 5, Female, 43 years)
	Patients taking on the burden of coordinating information between practitioners	Self-coordination of Care	‘I make sure that they speak to each other … everyone is informed.’ (Julie, Participant 3, Female, 53 years)
	Limitations because of public healthcare constraints affecting consistent care	Systemic Resource Constraints	‘If you’re in a state hospital … you can only do that much.’ (Keisha, Participant 6, Female, 38 years)
	Patients valuing continuity and consistency from healthcare providers who understand their history	Continuity of Care	‘During Covid … they didn’t know how to handle a Lupus patient.’ (Esther, Participant 4, Female, 35 years)

SLE, systemic lupus erythematosus; COVID-19, coronavirus disease 2019.

### Ethical considerations

The study adhered to all ethical guidelines as set out by the Social, Behavioural, and Education Research Ethics Committee (REC: SBE). Ethical clearance was obtained (Ethical Clearance Number: 24082), ensuring that all procedures met the necessary ethical standards. Key ethical considerations included informed consent, where participants were provided with an information letter and an informed consent form. They were given the opportunity to ask questions and withdraw from the study at any time without penalty. Participants’ identities were anonymised in all transcripts and reports to protect their privacy, with data stored securely and accessible only to the research team (Shaw 2003) A non-disclosure agreement (NDA) was signed by all research team members to ensure confidentiality (Yin 2013). Participants were also informed about the availability of counselling services if they experienced distress during the interviews. By adhering to these ethical principles, the study ensured that participants’ rights and well-being were prioritised throughout the research process.

## Results

The management of SLE requires a comprehensive approach that addresses the multifaceted nature of the disease. The findings of this study share three overarching themes identified through the analysis of participant interviews: *Collaborative Care, Patient Centredness, and Fragmentation Challenges*. The findings reveal the importance of a cohesive team of healthcare professionals, the necessity of centring care around participant experiences and the complexities within the healthcare system that create barriers to effective SLE management. In examining these themes, the findings highlight critical elements that can contribute to improving the quality of care for individuals living with SLE.

### Collaborative care

The participants consistently recognised the pivotal role of service providers in their treatment regimen considering each provider as part of their team. All participants felt that it was key that professionals had an aptitude for operating in interprofessional networks. Central to this collaborative relationship was the participant as patient serving as the focal point around which professionals were expected to coalesce. For participants, these professionals were directly connected to each other because of their efforts to treat SLE patients. This necessitated the cultivation of robust working relationships to optimise treatment outcomes for participants. Subsequently, participants employed multifaceted strategies to establish and sustain their medical teams. They felt that building a trusted support network and leveraging others’ professional expertise were more than an investment in their health; it enhanced their prospects and quality of life. Moreover, this approach was likened to management within complex organisational structures. Significantly, all participants noted that forming these teams was a gradual process, requiring careful consideration of the diverse impacts of SLE, including its physical and psychological effects on individuals:

‘The most successful people and conglomerates have people around them, a network. It’s a relationship, it’s a network and there’s elements of how you scale your network to be able to monetise it in a career, it’s similar with the disease. I’ve known I need people around me. I don’t have all the answers.’ (Ross, Participant 1, Male, 41 years)

Participants described a ‘required sensibility’ that was necessary from professionals and essential for the roles played by others in their healthcare journey. Professionals therefore needed to be receptive to a patient’s perspective as well as their professional peers. Significantly, the desire to be ‘heard’ and for professionals to be responsive rather than reactive was evident. Moreover, fostering opportunities for collective decision-making was raised and felt to contribute to reducing the fears and anxieties of patients. Where professionals accommodated this approach, participants experienced a heightened sense of stability, control and agency in the management of the disease:

‘My nephrologist – we know each other long. We’ve been through a few journeys. He’s one of a kind and brought stability – he is part of my team. If I make a decision, I talk to him. I will talk to him at least once a month. We’re on first name basis. If I message him now, he’ll reply. It’s very rare. He can put up with me and he’s taught to manage my disease. I’m fine for now, but there’s always that thing in the back of your mind, when is this thing going to come back.’ (Ross, Participant 1, Male, 41 years)

Selectivity in accepting care from medical professionals was crucial and discerned in consultation by the patient and their identified primary practitioner. This consultation invariably determined others’ entry into the care team. There was consensus from participants that a supportive and knowledgeable medical practitioner would positively affect a patient’s quality of care and their experience of the disease:

‘I messaged my rheumatologist and I said to her, ‘I’m going to be honest with you, he was terrible’. And she immediately said to me, ‘Then you’re not going back to him ever again’. You don’t need that. There are other doctors that are great, if that was your experience, he’s a brilliant doctor, but I’m not going to let him treat my patients and be dismissive.’ (Esther, Participant 4, Female, 35 years)

Treatment outcomes were improved and observable when medical practitioners had knowledge and working experience in managing lupus. These experienced professionals were characterised through a consistency in their messaging, the delivery of information with empathy and acknowledged the individuality in the experience of SLE:

‘As a Lupus person there are times you come into the ER and we’d rather wait to the next morning to phone our doctor to say, ‘Oh my gosh, I need to come in’. No offence to the ER doctor – it’s not their expertise. So, they tend to do all the tests they can do during that night and put on a drip and give you pain medication. I might as well wait for the next morning for my own doctor.’ (Julie, Participant 3, Female, 53 years)

The presence of these traits instilled greater confidence and trust between patient and professional and enhanced the efficacy of their treatment regimens. Professionals were expected to understand the interconnectedness of clinical proficiency, the desired interpersonal skills and their roles in improving health outcomes and patient satisfaction:

‘If I say to her, I have extreme pain or my chest is sore, she’s like, Okay we know this is how you present when you start becoming ill so let’s quickly get you on a drip for a weekend and you’re good to go. So, it’s having that team [*which is*] really important. It’s very hard to find the right doctors. A lot of people think they know something, but I don’t even bother going to a GP, I’m sorry. They can look after children and old people with flu. I don’t even waste my time to be honest.’ (Esther, Participant 4, Female, 35 years)

Geographic proximity improved transferring professional input and resulted in more comprehensive provision of care services. Working in the same hospital proved advantageous as it fostered incidental interactions among healthcare professionals reducing the time between the sharing of patient information and integration of services. Participants noted that proximity not only facilitated professional collaboration but also cultivated more meaningful relationships between patients and professionals:

‘I was diagnosed in ABC Hospital where I had a physician, Dr X, and my urologist, Dr Y and when I had the TIA’s Dr Z the neurologist was there. It was good when all of them were in one hospital. However, Dr X moved [*to another province*] to a university then it became more difficult then Dr Z moved to [*another suburb*]. Dr A I have to see her in another suburb and hospital and my urologist is in the urology hospital [*city centre*].’ Erica, Participant 5, Female, 43 (years)

Because of the complexities associated with SLE, participants expected a commitment to collective problem solving. In this context, collaboration was essential to improve the quality of care. Participants believed the quality of care was best achieved through case discussions aimed at consensus building between professionals followed by direct patient feedback. For participants, experiencing the consensus building was instrumental in enhancing their personal understanding of SLE:

‘Dr A is not in the X hospital he opened up his own space. Thursdays is a roundtable, it’s show and tell, “Right, this is what’s happening with Julie” and everybody gives input. Dr C will come back to me and say, “Listen, this is what this one’s seen this and that one’s seen that”. It was fantastic when everybody was at X hospital, they’d bump into each other [*daily*]. I’ve made it work cc’ing everybody for any blood test or scans.’ (Julie, Participant 3, Female, 53 years)

Although participants acknowledged the benefits of proximity and coordinated care, they were realistic with their expectations outside of private practice settings. Despite expertise available in state hospitals, participants felt they needed to temper their expectations concerning the quality of care available because of subpar administration and resource constraints that affected how professionals could provide care. Julie acknowledged the constant need to rebuild relationships with professionals in public hospitals and reshare information about her condition:

‘If you go to a state [*hospital*] the problem is not just being able to see the same doctor. I need to build a relationship with my doctors and in state I’m not going to have that relationship.’ (Julie, Participant 3, Female, 53 years)

In other instances, Carmen expressed her sadness of having to leave a public healthcare programme that she was accustomed to because of administrative challenges and more consistent access to her required medication:

‘The admin at government hospitals, I think God has specifically placed them there to teach us patience. I have stopped going because the medication, they have access to it’s not helping me anymore. They explained it would be better to be under [*Dr C*] because he’s got access other medications. It was actually a sad day.’ (Carmen, Participant 2, Female, 57 years)

While administrative issues affected participants’ experiences, Keisha acknowledged that because of the limited number of professionals available in public healthcare settings, collaboration among professionals would be limited. She considered the resource deficiency to affect her health outcomes:

‘If you’re one doctor, with three staff members with a patient turnover of 100 within an hour, you can only do that much. If you don’t have the medication and the equipment to treat the patient, you can only do that much. They do an excellent job, but they don’t always have what they need. I’ve got immense respect for people in the public sector, but I don’t want to be a patient because they don’t have the means to do what they need to do.’ (Keisha, Participant 6, Female, 38 years)

Advocacy and educating others was perceived as part of their personal role as a team member. Through these efforts, they could actively improve the understanding and promote the need for better care for patients with less visible chronic diseases. Participants became increasingly aware of the systemic challenges within healthcare and took on the role of educator to promote the awareness of their experiences:

‘I was admitted to a [*government hospital*] for an infection in my bladder and the nurses said, “Okay, doctor has given you strong pain medication and antibiotics, but you look very well. Please explain this?” She couldn’t understand why I have a catheter and seem okay. I put up fliers and asked for 10 minutes when all the nurses get together and explained Lupus is an autoimmune disease. The nurses took it to the hospital manager, and she then said, “This is actually so nice, let’s send it to all the staff.”’ Erica, Participant 5, Female, 43 (years)

### Patient centredness

Effective strategies for managing chronic illnesses hinged on placing the patient at its centre, ensuring accessibility, convenience and regularity of contact with healthcare providers who were empathetic. Here, technology-mediated communication emerged as a valuable asset in the healthcare process. Utilising platforms like WhatsApp and video calls facilitated remote medical assistance, particularly beneficial to participants experiencing mobility barriers. Significantly, participants highlighted that not only did this direct access offer emotional and psychological support but also contributed to an enhanced healthcare experience centring them instead of their treatment regimes:

‘My therapist lives just and works from home or we’re online. If I need a face to face, I can go to coffee with her at her home. I can see her wherever. I would have WhatsApp calls, video calls … it’s easy for me not to leave home … You need that other person to speak to [*who*] also plays a huge role in my life.’ (Julie, Participant 3, Female, 53 years)

The clarity with which information was delivered was also crucial for patients to comprehend the intricacies of the disease and the trajectory of their treatment. Participants called for communication balanced with a mix of transparency, empathy and optimism between doctor and patient when explaining the gravity of the impact of SLE and determining a course of action:

‘Not my GP. I came to know more about Lupus from the rheumatologist at the State Hospital. He takes time to explain everything and put me on new medication. He specifically told me not to Google the side effects with new medication because then I would probably not drink it.’ (Carmen, Participant 2, Female, 57 years)

When clarity and transparency were evident, participants were better equipped to moderate their expectations and manage the anxieties associated with their condition. Communicating in this truthful manner created a supportive environment and avoided the reliance on other resources and information sources, which deterred them from receiving treatment:

‘It was awful but he explained it to me very well. “This is going to be a long journey, and a few people go into remission. Don’t get your hopes up, right now with going to go into remission. Don’t listen to people that you can be healed. Just realise this is where you’re going.”’ (Julie, Participant 3, Female, 53 years)

Participants highlighted that SLE typically manifests gradually and exhibits characteristic symptoms, which required proactive management and the meticulous monitoring of symptoms over prolonged periods:

‘We get to know our bodies so I will tell them, week one, “Something’s brewing” by week 3, I say, “Right, now I’m coming, I need to see you, and then I have an infection and whatever. So, it’s important to have that team and I advise people to make sure they have a team of doctors that actually know each other.’ (Julie, Participant 3, Female, 53 years)

As time progressed, all participants experienced a deepening self-awareness and understanding of their symptoms and the severity. Consequently, health literacy and patient autonomy emerged as pivotal components in the ideal client-centred approach to lupus management:

‘I had an idea about it. I didn’t really understand what was in store for me.’ (Esther, Participant 4, Female, 35 years)

‘There are patients still afraid, when they hear doctor, they feel they can’t question. I’m past that. I will question and you need to give me answers that I need.’ (Julie, Participant 3, Female, 53 years)

Those who actively pursued knowledge felt empowered to challenge the hierarchical practitioner-patient dynamic to make more informed decisions about their care. For participants hospitalised regularly, their confidence in navigating healthcare resources improved with the increasing familiarity. This was further developed as professionals gained insights into their patients’ needs and shared their valuable insights into the healthcare system for their patients to benefit:

‘I know my symptoms. I’m a dictator when sick. I decide when I’m going to hospital, nobody else tells me. I know if I can heal at home or the hospital. I know how to work the medical system…use the hospital element of your medical [*aid*] and go in for three days, it’s like a hotel essentially for me. The relationship that I had with my doctor helped.’ (Ross, Participant 1, Male, 41 years)

Increased efforts to navigate the system during critical times heightened their vulnerability, given their compromised immune system. The coronavirus disease 2019 (COVID-19) pandemic disrupted specialised care significantly amplifying the risks of poor health outcomes and necessitated persevering under these conditions:

‘Covid hit. My Nephrologist, an elderly professor, did not want to see any patients or admit anyone to hospital. It’s like you’re safer out of hospital. No one could figure out what to do with me. Luckily I found a rheumatologist who eventually figured out after three months and started me on medication. She’s my primary doctor now.’ (Esther, Participant 4, Female, 35 years)

Resilience emerged prominently in all participants’ narratives in relation to engaging with the healthcare system. For most participants, the simplest way to overcome this was to enter into private healthcare at a substantial personal cost. Carmen and Julie expressed a similar concern:

‘When you are at government hospitals, the months you don’t have to see a doctor you still have to collect your medication. That means another day, to sit at a clinic. Privately I can go on a Saturday and my script which was six months, I collect it from the pharmacy. I’m not going to take off work as under the government treatment. At a private doctor it’s easier because it doesn’t influence your work except if you fall ill, yes.’ (Carmen, Participant 2, Female, 57 years)

Julie in turn suggested that the direct and indirect burden of SLE rose in such circumstances. Resilience therefore was a higher priority when receiving care through state hospitals compared to private facilities. The necessity for financial, emotional and physical resilience particularly in navigating the availability (or lack thereof) of essential medications rose for all participants:

‘With state hospitals, if they start you on [*medication*], they have to make sure they have that product in stock for the next visit … the state runs up a bill and pharmaceutical companies get fed up and go, “right, enough now. Pay your bill and then we will give you meds.” Then someone comes and their biologics is not available. Can’t have that – it has to be available.’ (Julie, Participant 3, Female, 53 years)

### Fragmentation challenge

Participants stressed the importance of coordinated care provided by cohesive healthcare teams. They sought assurance that professionals shared a unified understanding of their diagnosis and treatment:

‘I’ve got a specialist physician then a general practitioner then a psychologist and a psychiatrist. I struggle most with [*getting*] all of my medical professionals to communicate with one another.’ (Keisha, Participant 6, Female, 38 years)

All participants advocated for a collaborative approach in formulating a personalised treatment plan tailored to their individual needs. The presence of interprofessional collaboration facilitated more comprehensive care delivery experiences, while participants identified timing issues between tests, diagnosis, feedback and treatment as major impediments. Participants emphasised the significance of reducing delays impacting their quality of life, particularly when professionals were unaware of conflicting treatment outcomes especially the interaction between medications prescribed for different conditions:

‘The problem when I have a urological problem like my stents, then Dr A would say, they have to remove the stents because that’s what’s causing the infection and then Dr B will tell me they cannot remove the stents because without the stents you’re not going to drain, then your kidneys are going to be zero. So that’s a bigger problem to get them to talk to each other.’ (Erica, Participant 5, Female, 43 years)

Conflicting medical care approaches surfaced when professionals lacked direct communication with each other. In response, participants took the initiative in directing and actively established channels for sharing information specifically to eliminate the communication barriers between professionals. However, this was time consuming and added to the burden and fatigue experienced by participants, highlighting the emotional and physical toll of managing such a disease. In navigating these systemic challenges, participants wanted more centralised care coordination and a designated practitioner responsible for consolidating medical opinions and reports. This would mitigate contradictory treatment plans, alleviate the burden on patients and enhance overall care coordination and outcomes:

‘Trust me, I make sure that they speak to each other. When I go to [*pathology laboratory*], they giggle because they know, “Oh, we need to CC this doctor, that doctor” and then they all get it because nowadays they get it on their phones, and they have a quick look, and everybody is [*then*] well informed as to what’s going on Julie.’ (Julie, Participant 3, Female, 53 years)

Participants also disclosed that the difficulty in having to coordinate their care among healthcare professionals reflected poorly on their interprofessional communication and ultimately impacted their treatment efficacy further ‘burdening’ them. They frequently encountered systemic issues when there was no established protocol for information sharing among professionals treating them leaving them to bridge this gap. Addressing issues such as fragmentation, conflicting opinions, resource constraints and systemic inefficiencies compelled participants to take on this mediating role in managing their care:

‘Normally, a bit of time goes into make sure doctors have the latest results and diagnosis done by other doctors, and then it’s not being communicated from the doctors to one another. Treatment interacts with one another, and they need to be aware of it. But they don’t communicate as much as I would like them to communicate with one another.’ (Keisha, Participant 6, Female, 38 years)

Continuity and consistency of care were also crucial for effectively managing lupus. Participants recognised that they could influence both factors by actively managing the flow of information and communication regarding their treatment:

‘I’ve always had good fortune to speak to doctors that have been prepared to listen, except during Covid with a shortage of doctors, and hospitals on lockdown. That was bad, because it was doctors that weren’t my usual team, and did not know how to handle a Lupus patient.’ (Esther, Participant 4, Female, 35 years)

Participants felt that the attunement to their own bodies contributed to developing their self-sufficiency and wanting control over their care-related information. However, this inclination often stemmed from the frustration of having to educate some healthcare professionals because of the rarity of their condition. Additionally, participants felt preparation and adaptability by professionals were critical for improving their treatment experiences. Participants felt that the unpredictable nature of the illness, along with the associated uncertainties added complexity to their treatment plans needing flexibility:

‘After three weeks in hospital, I was tired and couldn’t bother to explain and said, “Okay, so Dr A you are typing up a report so I can give it to my other two doctors and Dr B give me a report on all medicine and things you think I need to take” and I took it to Dr C – now my main doctor. So, between the two she gets reports from them, so she knows why they’re doing certain things.’ (Erica, Participant 5, Female, 43 years)

For all participants, the management of SLE hinged on the ability to foster collaborative care teams, ensure client-centred approaches and navigate the challenges of healthcare fragmentation. Participants benefitted significantly from coordinated care where professionals communicate effectively and work together to create personalised treatment plans. Placing the patient at the centre of care, utilising technology for better communication and ensuring that patients are well informed and empowered are crucial strategies. Addressing the systemic issues of fragmentation and conflicting medical opinions required improved coordination and a more centralised approach to care management. By focusing on these areas, the overall healthcare experience and outcomes for individuals with lupus can be enhanced, ensuring they receive the comprehensive and empathetic care they need.

## Discussion

The findings of this study provide insight into the management of SLE, particularly regarding the critical roles of collaborative care, patient centredness and the challenges posed by healthcare fragmentation. These findings align with existing literature but also contribute new perspectives that are relevant to the ongoing research and discourse on chronic illness management.

### Collaborative care

The study highlights the importance of cohesive healthcare teams in effectively managing SLE. Participants repeatedly emphasised the necessity of having a trusted network of healthcare providers who are knowledgeable and experienced in the complexities of SLE and capable of functioning within interprofessional networks. This finding is consistent with the literature on IPCP advocating for cohesiveness from professionals and acknowledging the importance of patients’ involvement in determining their care (D’Amour & Oandasan 2005). Moreover, this study highlights the challenges within public healthcare where resource constraints impact patient outcomes (Wranik et al. 2019) and the importance of proximity or working in shared spaces (Dahl & Crawford 2018; Sangaleti et al. 2017). Moreover, the applicability to MDTs and IDTs managing chronic conditions is also evident (Farina et al. 2022; Soukup et al. 2018). The study adds to this body of knowledge by demonstrating how the lack of collaboration can lead to fragmented care, delays in treatment and suboptimal patient outcomes.

### Patient-centredness

The emphasis on client-centred care in the study aligns with the broader movement towards SDM and the integration of PROMs in chronic disease management (Légaré & Witteman 2013; Makhni & Hennekes 2023). The participants in this study valued clear, empathetic communication from their healthcare providers and appreciated the use of technology, such as WhatsApp and video calls, which facilitated remote consultations and provided emotional support. These findings suggest that when patients are placed at the centre of their care, with consistent and empathetic communication, they are more likely to be engaged in their treatment plans and experience better health outcomes. This is particularly important in the context of SLE, where the variability of the disease requires personalised care plans that are responsive to the patient’s unique needs.

### Fragmentation challenges

One of the most striking findings of this study is the extent to which fragmentation within the healthcare system impacts the care of individuals with SLE. Participants frequently encountered delays, conflicting medical opinions and the burden of coordinating their care, all of which detracted from their overall healthcare experience. This issue of fragmentation is well documented in the literature, where it is associated with poorer health outcomes (Hossny et al. 2022). This study contributes to the discourse through providing specific examples of how fragmentation manifests in the care of SLE patients and by highlighting the need for more integrated care models that can reduce these barriers. By addressing these systemic issues, healthcare systems can improve the quality of care for SLE patients, ensuring that they receive timely, coordinated and effective treatment.

Overall, the findings’ emphasis on collaborative care and patient-centredness aligns with practices in the field, while the identification of fragmentation challenges adds a critical dimension to the discourse. These findings not only confirm existing theories but also provide new insights that can inform future research and practice in the management of SLE and other complex chronic conditions.

## Conclusion

This study highlights the importance of a collaborative, client-centred approach to managing SLE while also emphasising the challenges posed by fragmentation within the healthcare system. By fostering strong interprofessional networks, utilising technology to enhance patient engagement and improving care coordination, healthcare providers can significantly improve the quality of care for individuals living with SLE. These findings have important implications for both clinical practice and future research, particularly in the development of integrated care models that promote collaboration among medical specialities and can better meet the needs of patients with complex chronic conditions.

Hospitals and clinics should invest in the implementation of advanced EHRs to enhance communication and reduce care fragmentation. Additionally, investing in training programmes that strengthen teamwork, communication and leadership skills within multidisciplinary teams is essential for improving patient outcomes.

With respect to the technology industry, it has a significant opportunity to innovate in telehealth platforms and mobile health applications. These technologies can bridge communication gaps and provide chronically ill patients with accessible, real-time support from their healthcare teams. Health tech companies should focus more intently on developing user-friendly tools that empower patients in managing their care while facilitating better coordination among healthcare professionals.

There are considerable opportunities for the pharmaceutical sector to play a more crucial role in ensuring the availability of medications for chronic conditions like SLE within the public healthcare system. Streamlining the supply chain and collaborating with healthcare providers to maintain consistent access to essential drugs are vital. Additionally, pharmaceutical companies should focus on educating both patients and providers about the latest treatments, emphasising a client-centred approach to care.

Policymakers should also incorporate these findings into healthcare policies, particularly in resource allocation and funding for chronic disease management. Strengthening public healthcare systems to support integrated care models is crucial. Policies that encourage ongoing education for healthcare providers and foster interdisciplinary collaboration will improve patient outcomes and reduce disparities.

The study’s limitations include a relatively small, purposive sample, which, while providing in-depth insights, may not capture the full range of experiences among individuals with SLE in diverse healthcare settings. Additionally, this research focuses on patient narratives within a specific geographic and systemic context, limiting the generalisability of findings to other healthcare systems or regions with different structural and cultural dynamics. Future research could expand by incorporating larger, more varied samples and investigating the impact of specific interprofessional communication practices on chronic illness management outcomes. Comparative studies between different healthcare systems could also shed light on structural factors that promote or hinder effective collaboration in chronic disease management, offering a broader understanding of best practices for interprofessional care.
